# Harmful Effects of Synthetic Surface-Active Detergents against Atopic Dermatitis

**DOI:** 10.1155/2015/898262

**Published:** 2015-01-15

**Authors:** Hajime Deguchi, Riho Aoyama, Hideaki Takahashi, Yoshinari Isobe, Yutaka Tsutsumi

**Affiliations:** ^1^Fujita Health University School of Medicine, Toyoake, Aichi 470-1192, Japan; ^2^Department of Pathology, Fujita Health University School of Medicine, Toyoake, Aichi 470-1192, Japan; ^3^Isobe Clinic, Anjo, Aichi 446-0026, Japan

## Abstract

We report herein two cases of intractable atopic dermatitis successfully treated by simply avoiding the contact with surface-active detergents in the daily life and living. The detergents were closely related to the exacerbation and remission of the disease. Steroid ointment was no longer used. We discuss that the removal of horny layer lipids by surface-active detergents accelerates the transepidermal water loss and disturbs the barrier function of the epidermis and thus is intimately involved in the pathogenesis of atopic dermatitis.

## 1. Introduction

Atopic dermatitis is etiologically related to abnormalities in physiologic functions of the skin, resulting in chronic persistent and irritating inflammation of type I and/or type IV allergic reactions: atopic dermatitis is a disease of altered epidermal barrier [1–4]. Allergens are usually not specified. Infants aged below 2 years show the lowest epidermal barrier function and are susceptible to atopic dermatitis [1–3]. The main victims are thus infants and young children, but the long-lasting disease is also seen in the adulthood. The treatment strategy against atopic dermatitis includes the external use of steroids or tacrolimus ointment, in addition to moisturizing and protective skin cares [5]. Internal use of antihistamines and antiallergic drugs and the elimination of exacerbating factors are also employed.

Dry skin is one of the major symptoms in atopic dermatitis. The abnormality of the epidermis, especially the horny layer (stratum corneum), is closely linked to loss of the barrier function. The transepidermal water loss is caused by reduced lipids in the horny layer. The lipid bilayers intermit between the horny keratinocytes (corneocytes). When the corneocytes are thought of as bricks, the lipids filling the spaces between the cells are the mortar or cement (brick and mortar model) [2, 4]. The lipid bilayers consist of ceramides, cholesterol and long-chained fatty acids, and impede penetration of lipophilic as well as hydrophilic substances [6–9]. Soap and detergents acting as surfactants may provoke skin damage such as scaling, dryness, tightness, roughness, erythema, and swelling. An itch-scratch cycle accelerates damaging the epidermal barrier [1–4].

We report herein two representative adult patients who showed exacerbation of atopic dermatitis after the contact with surface-active detergents and the disuse led to the remission. We propose that the removal of horny layer lipids by surface-active detergents is intimately involved in the pathogenesis of atopic dermatitis, as one of the authors have published Japanese-written books for promoting the general public and dermatitis patients to avoid using soap and detergents [10, 11].

## 2. Case Presentation

**Figure 1:**
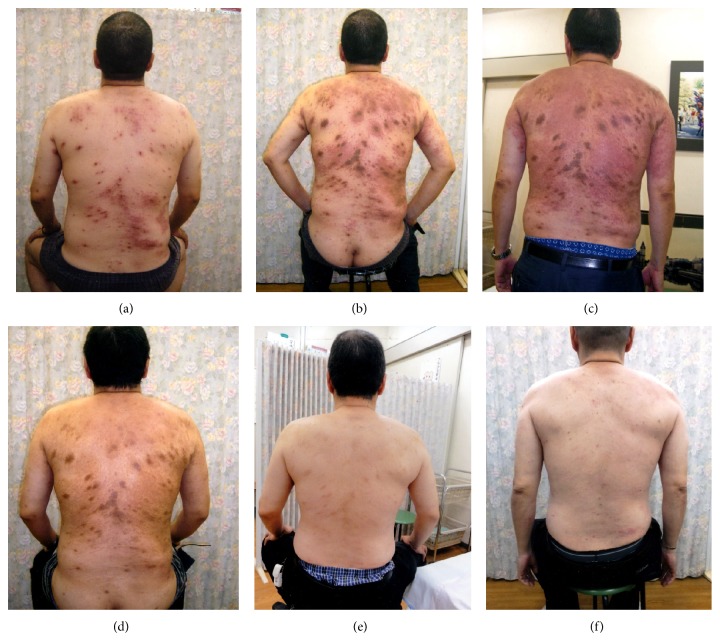
Clinical features of skin rash on the back in case 1 (a 50-year-old male). (a) December 4, 2010 (the first medical inspection), (b) January 12, 2011 (exacerbation), (c) January 26, 2011 (the worst state with erythematous reaction), (d) March 16, 2011 (alleviation), (e) July 17, 2012 (remission), and (f) March 11, 2014 (recurrence). Strict avoidance of the detergent-containing material and usage of cleansing soap without detergents were quite effective to control atopic dermatitis.

*Case 1*. Case 1 is a 50-year-old male, an office worker in a gas station. After a 10-month history of chronic prurigo treated with steroid ointment, he visited Isobe Clinic in Anjo, Aichi, Japan, in November, 2010. He complained of itchiness all over his body, resulting in difficulty in sleeping. Based on the chronic and repetitive rash with itchy sensation, the diagnosis of atopic dermatitis was made (Figure 1(a)).

**Figure 2:**
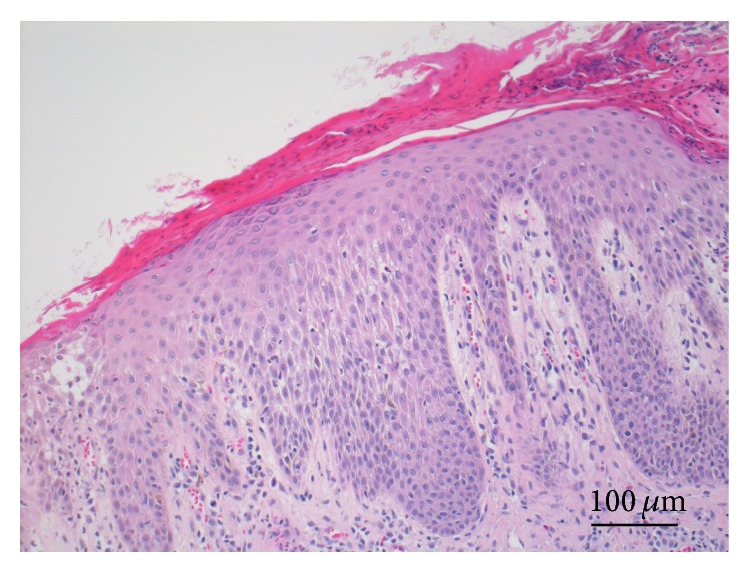
Microscopic features of the biopsied abdominal skin in case 1 (hematoxylin and eosin). The involved epidermis reveals reactive downward acanthosis with lymphocytic exocytosis and spongiotic reaction focally resulting in small vesicle formation. An eosinophilic microabscess is formed in the parakeratotic horny layer. The granular keratinocytes have disappeared. Superficial perivascular infiltration of lymphocytes and eosinophils is associated.

With the radioimmunosorbent assay for allergens in January, 2011, no specific antiallergens were identified in the serum. A total of 12 allergens were evaluated, including Japanese mugwort, house dust, house dust mite (*Dermatophagoides pteronyssinus*),* Alternaria* (air-floating black fungus), egg white, pork, shrimp, mackerel, cone, rice, buckwheat, and peanut. The serum IgE level was not significantly high, 189 IU/mL (normal range: ~170 IU/mL). Repeated bacterial culture tests performed four times during November, 2010, through April, 2013, failed to detect any specific pathogens. The biopsy was performed from his right abdominal skin. The microscopic findings are illustrated in Figure 2. Reactive downward acanthosis with lymphocytic exocytosis and spongiotic reaction focally resulting in small vesicle formation is shown. An eosinophilic microabscess was formed in the parakeratotic horny layer. The granular keratinocytes disappeared. Superficial perivascular infiltration of lymphocytes and eosinophils was associated.

The patient was asked to avoid using synthetic surface-active detergent-containing material such as cleansing soap, household synthetic detergents, shampoo and conditioner, and cosmetic cream and lotion. The use of natural soap was also avoided. When bathing, the hair and body were washed only with warm or tepid water. The skin was cared with an ointment consisting of a mixture of vaseline and urea (urea concentration: 0.12%). Concurrently, the following drugs were prescribed: (A) Celtect (Oxatomide), 2 tablets (antiallergic drug), (B) Nipolazin (Mequitazine), 2 tablets (antihistamine), (C) Tarivid (Ofloxacin), 2 tablets (new quinolone antibiotics), and (D) Terramycin ointment (Tetracycline antibiotics). When necessary, Amikacin (aminoglycoside antibiotics) was intramuscularly injected. The antibiotics were administered because of the clinical suspicion of coinfection of anaerobic bacteria.

His skin condition was not significantly improved soon, and the rash was exacerbated in January, 2011 (Figure 1(b)).  Figure 1(c) demonstrates the worst state of his erythematous rash on the back, photographed 10 days after Figure 1(b). One of the reasons for the exacerbation was considered to be linked to the fact that the cosmetic companies recently increased the concentration of synthetic surface-active agents in their products, including shampoo, hair conditioner, and synthetic cleaning soap. The patient was again advised to avoid strictly contacting with the detergent-containing material and using soap without detergents “Bajan” (soap prepared by electrolysis of sodium bicarbonate water, Kenbi, Iwate, Japan) for washing clothes. Two months later, the skin rash was improved with much less itchy sensation (Figure 1(d)). Thereafter, the avoidance strategy effectively alleviated the condition of his skin. One and half years later, the skin rash was controlled completely (Figure 1(e)), and his symptoms including itchy sensation disappeared and he became able to sleep well.

In March, 2014, itchy skin rash recurred, because his wife started to use the detergent-containing synthetic cleansing soap, which is widely used in Japan. On inspection, small-sized rash accompanied by itchy sensation was observed on his back (Figure 1(f)). For the symptomless two-year period, he and his wife believed that his atopic dermatitis has been cured completely. Reeducation of the patient of his wife was necessary to improve his skin condition. In March, 2014, his serum IgE level remains low as 59 IU/mL.

*Case 2*. Case 2 is a 48-year-old female, a housewife. The diagnosis of atopic dermatitis was made when she was a junior high school girl. In order to control the skin rash, steroid ointment was administered for some 40 years. The control status was not excellent, and she occasionally complained of itchiness all over the body. In June, 2007, she met one of the authors (Y. I.) who gave a lecture on how to treat and control atopic dermatitis. Y. I. personally delivered an ointment containing dibucaine and hydrophilic vaseline, which significantly relieved her itchy sensation.

**Figure 3:**
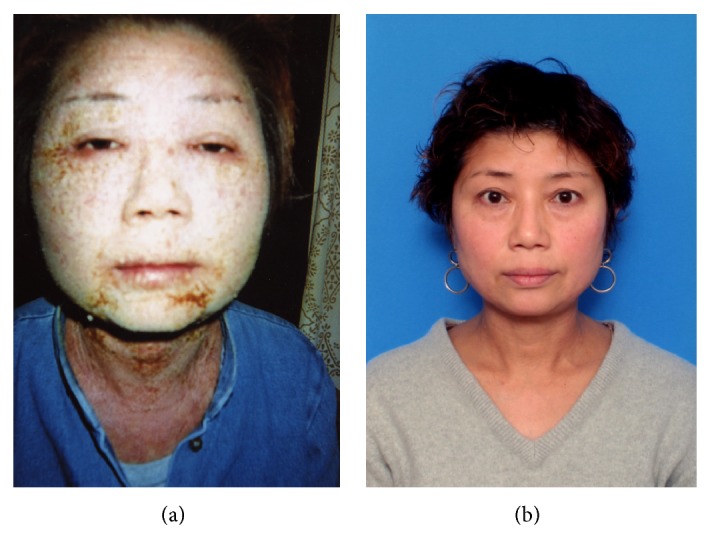
Clinical features of facial skin rash in case 2 (a 48-year-old female). (a) October, 2007 (severe rash), (b) June, 2014 (complete remission). After the patient happened to use shampoo and body soap equipped in a hotel, her face became markedly swollen by severe and itchy rash with secondary infection and scratch injury. Complete avoidance of the detergents and cleansing soap led to long-lasting complete remission without using steroid ointment. The patient allowed us to present her whole face.

When her skin rash was under control by the topical use of steroid subscribed from a university hospital, she happened to use shampoo and body soap equipped in a hotel. Thereafter, her face became markedly swollen by severe and itchy rash with secondary infection and scratch injury (Figure 3(a)). She thought that this event occurred as a rebound phenomenon of steroid therapy. Finally, the patient visited Isobe Clinic in October, 2007, and she was advised to avoid using synthetic surface-active detergent-containing material such as cleansing soap, household synthetic detergents, shampoo and conditioner, and cosmetic cream and lotion, and the skin was coated with an ointment containing a mixture of vaseline and urea. The use of natural soap was also avoided. Concurrently, the following drugs were prescribed: (A) Nipolazin (Mequitazine), 2 tablets (antihistamine), (B) Chloromycetin salve (antibiotics), (C) Tarivid (Ofloxacin), 2 tablets (antibiotics), (D) Cinal, 4 tablets (vitamin compounds), and (E) Depas05, 2 tablets (antianxiety drug). Azunol ointment (anti-inflammation drug) was also used when necessary.

By the end of November, 2007, her skin condition improved dramatically (Figure 3(b)). Thereafter, she continued to avoid thoroughly using the detergent and soap. In July, 2014, the condition of her skin was kept well without steroid therapy any longer.

## 3. Discussion

We report herein two representative adult cases of atopic dermatitis, against which the avoidance of synthetic surface-active detergent-containing materials such as cleansing soap, household synthetic detergents, shampoo and conditioner, and cosmetic cream and lotion was quite effective in relieving the symptoms and signs. The patients were also asked to avoid using natural soap. Activity of atopic dermatitis was histologically evident in the biopsied skin of case 1. Parakeratotic changes accompanied by disappearance of the granular keratinocytes directly represented epidermal barrier dysfunction. Reuse of the detergent-containing material exacerbated the skin condition. Supportive therapy included topical rubbing of an ointment containing a mixture of vaseline and 0.12% urea and administration of antihistamines, antiallergic drugs, and antibiotics. Steroid ointment was no longer used in these two cases. Such a cost-effective treatment strategy dramatically improved the condition of long-lasting and intractable atopic dermatitis. It is evident clinically that the synthetic surface-active detergent caused the exacerbation of atopic dermatitis.

One of the authors, Yoshinari Isobe, M.D., is a practical dermatologist in Anjo, Aichi, Japan, having long and deep experience of the cost-effective treatment against severe and refractory atopic dermatitis. He has promoted patients of atopic dermatitis and the general public not to use the material containing surface-active detergents in the daily life and living. Based on the clinical experience treating more than 400 adult cases of intractable atopic dermatitis, he published promoting books for the general public and dermatitis patients, written in Japanese [10, 11]. He insists that complete avoidance of the detergent results in complete remission of atopic dermatitis.

We propose that the removal of horny layer lipids by the surface-active detergent is closely related to the pathogenesis of atopic dermatitis. The representative surface-active agent in the commercially available synthetic soap is polyoxyalkylene alkyl ether, an active emulsifier and detergent for cosmetics, general cleaner, emulsifier for emulsion polymerization. Polyoxyethylene lauryl ether, an emulsifier for cosmetics, is also commonly added (quoted from the ingredient labeling). The detergent takes the lipid component of the epidermal horny layer away and disturbs the barrier function of the epidermis. The transepidermal water loss is caused by the reduction of lipids in the horny layer. The horny keratinocytes (corneocytes) are known to be intermitted by the lipid bilayers, consisting of ceramides, cholesterol, and long-chained fatty acids. The lipid actively secreted from lamellar granules of the granular layer keratinocytes undergoes enzymatic processing to produce the lipid bilayers [6–8]. According to the brick and mortar model [2, 4], when the corneocytes are thought of as bricks, the lipids filling the spaces between the cells represent the mortar or cement.

The flattened corneocytes are interlocked by specially strengthened desmosomes with each other. The long chained ceramides ensure the cohesion of the lipid bilayers between the corneocytes. In other words, the corneocytes sealed in the lipid secretions form the insoluble and fluid impermeable surface coat. These structures give the physiologic stability of the horny layer [6–9]. The corneocytes are devoid of the cell organelles and nucleus but are still metabolically active. Hydrolases hydrolyze triglycerides into di- and monoglycerides, and proteases ensure the supply of amino acids in order to maintain the natural moisturizing factor from proteins [2, 4, 12]. It is known that the use of soap and detergents results in the elevation of horny layer pH and that the sustained increase in pH enhances the activity of degradatory proteases and decreases the activity of lipid-synthesizing enzymes [2, 4]. Normal flora may contribute to skin surface homeostasis, and this sensitive balance is disturbed by the external use of inappropriate hygienic material and cosmetic products [3, 13].

Filaggrin, the key superficial epidermal component of keratinization and lipid secretion, is cleaved from profilaggrin, a major basic protein of keratohyalin granules of the granular layer keratinocytes. Filaggrin binds to and condenses keratin cytoskeleton in the corneocytes and is citrullinated to function as a natural moisturizing factor [14]. Abnormalities of the filaggrin gene are seen in some patients with atopic dermatitis [2, 4, 15, 16]. The importance of the lipid bilayers in the epidermal frontline should again be emphasized.

The transepidermal water loss is especially important in the barrier damage. Not only natural soap, household synthetic detergents, shampoo, and conditioner but also emulsifiers in creams or lotions and tensides in cleansing products contain surface-active substances and damage or even destroy the intercellular lipid bilayers. Loss of the lipophilic component out of the lipid bilayers increases transepidermal water loss, resulting in skin dehydration (dryness). The dysfunction of the lipid bilayers accelerates the diffusion and permeation of irritable water soluble substances into the deeper part of the epidermis. Topically applied occlusive substances such as urea-containing vaseline prevent the transepidermal water loss. Recently, barrier-restoring or ceramide-replacing therapies have been proposed for atopic dermatitis [17, 18].

We would like to emphasize the possibility of cost-effective and steroid-free therapy of intractable atopic dermatitis simply by avoiding the contact with the surface-active detergent in the daily life and living.
